# Longitudinal Imaging Patterns Following an Imaging‐Guided Dual‐Axis Picosecond Nd:YAG Protocol for Facial Hyperpigmentation

**DOI:** 10.1111/jocd.71155

**Published:** 2026-08-20

**Authors:** Sung Joo Kwon, Ji Yu Bang

**Affiliations:** ^1^ Toxnfill Clinic Jeju Republic of Korea; ^2^ Vands Clinic Jeju Republic of Korea

**Keywords:** dual‐axis protocol, facial hyperpigmentation, multimodal skin imaging, picosecond Nd:YAG laser, polarized‐light imaging, quantitative image analysis

## Abstract

**Background:**

Facial hyperpigmentation is commonly treated with pigment‐directed laser procedures and repeated low‐fluence toning. Although effective in many patients, these approaches may be followed by post‐inflammatory hyperpigmentation (PIH), recurrence after initial improvement, or therapeutic plateaus that limit further pigment clearance.

**Objectives:**

To characterize longitudinal multimodal imaging patterns following an imaging‐guided dual‐axis picosecond Nd:YAG protocol and to determine whether changes in pigment‐associated measures were accompanied by changes in polarized‐light (PL) imaging.

**Methods:**

This retrospective, nonrandomized, hypothesis‐generating study included 53 patients with persistent or recurrent facial hyperpigmentation characterized by coexisting epidermal‐predominant and dermal‐predominant pigmentary components on multimodal imaging. The dual‐axis protocol group (*n* = 15) received selective 532‐nm irradiation of epidermal‐predominant pigmentation and repeated 1064‐nm irradiation of dermal‐predominant or mixed lesions, with the 1064‐nm component calibrated primarily according to transient focal erythema within a clinically acceptable sub‐purpuric range. The retrospectively identified conventional‐treatment group (*n* = 38) received heterogeneous multimodal laser treatments reflecting routine clinical practice. Standardized lesion‐centered regions of interest were analyzed at baseline, Session 5, and Session 9 using normal‐light, ultraviolet (UV), and PL imaging. Outcomes included the Melanin Index (MI), UV index, and PL index.

**Results:**

At Sessions 5 and 9, the dual‐axis protocol group showed greater reductions in MI, UV index, and PL index than the conventional‐treatment group. At Session 9, median percentage reductions were 43.7% for MI, 40.6% for UV index, and 18.0% for PL index in the dual‐axis protocol group, compared with 9.1%, 8.0%, and 2.6%, respectively, in the conventional‐treatment group. Baseline‐adjusted differences were significant for all three indices (all *p* < 0.001). Complete‐case analysis, sensitivity analysis addressing baseline UV imbalance, and single‐index responder analyses yielded directionally consistent findings. In 15 selected cases, two manual intra‐observer reassessments showed directional concordance of 94.4% and 96.7% with the original analysis.

**Conclusions:**

The dual‐axis protocol was associated with coordinated longitudinal reductions across pigment‐ and PL‐associated imaging domains. These exploratory findings provide a rationale for prospectively evaluating whether multimodal imaging, including normal‐light, UV, and PL imaging, can assist treatment‐target selection, serial reassessment, and response‐calibrated treatment. They do not establish clinical utility, vascular–melanocytic decoupling, or causal treatment superiority and require validation in prospective controlled studies.

## 1 Introduction

Facial hyperpigmentation has traditionally been treated with pigment‐targeted laser procedures [1, 2, 3, 4] and repeated low‐fluence laser toning [3, 5]. Although these approaches are widely used and clinically effective in many patients, real‐world practice frequently encounters post‐inflammatory hyperpigmentation (PIH), recurrence after initial improvement, and therapeutic plateaus in which further pigment clearance becomes limited [3, 5, 6, 7]. These observations suggest that additional treatment strategies warrant exploration in patients who do not achieve sustained improvement with conventional pigment‐centered approaches.

Recent studies on the pathophysiology of pigmentary disorders suggest that melanocyte activity may be influenced not only by residual melanin burden but also by persistent upstream signals arising from the epidermis, inflammatory processes, and the vascular compartment [8, 9, 10, 11, 12]. From this perspective, persistent or recurrent hyperpigmentation may reflect a local tissue environment that continues to support melanogenic activity even after partial pigment clearance [7, 11, 12]. This provides a biologically plausible rationale for exploring strategies that address epidermal, inflammatory, and vascular‐associated influences alongside residual pigment burden, rather than focusing exclusively on repeated pigment‐directed treatment [5, 7, 11, 12].

In the present study, we evaluated longitudinal imaging changes following an imaging‐guided dual‐axis picosecond Nd:YAG protocol. The protocol combined selective 532‐nm irradiation of epidermal‐predominant pigmentary components identified clinically and on multimodal imaging with repeated 1064‐nm irradiation of dermal‐predominant or mixed pigmentary components. Potential biological interpretations were considered only as hypothesis‐level models for interpreting the observed clinical and imaging findings.

Using standardized lesion‐centered region‐of‐interest (ROI)–based quantitative imaging, we compared longitudinal changes in the Melanin Index (MI), ultraviolet (UV) index, and polarized‐light (PL) index between the dual‐axis protocol group and a retrospectively identified conventional‐treatment group. We sought to determine whether reductions in pigment‐associated indices were accompanied by longitudinal changes in PL‐associated optical patterns and to explore the relevance of multimodal imaging for longitudinal treatment assessment. Accordingly, this retrospective study was designed to generate hypotheses for future prospective investigation.

## 2 Methods

### 2.1 Study Design and Patient Population

This retrospective, nonrandomized, hypothesis‐generating study analyzed longitudinal imaging data from 53 patients with facial hyperpigmentation treated at a single clinic. Two real‐world treatment cohorts were compared, with the conventional‐treatment group serving as a retrospectively identified comparator.

All patients who met the eligibility criteria and had available longitudinal imaging data during the respective treatment periods were included. Patients were classified post hoc according to the treatment strategy received independently of subsequent clinical or imaging outcomes.

Eligible patients had persistent or recurrent facial hyperpigmentation with coexisting epidermal‐predominant and dermal‐predominant or mixed pigmentary components identified on standardized multimodal imaging. Clinical diagnoses included mixed melasma, acquired bilateral nevus of Ota‐like macules (ABNOM), solar lentigines, ephelides, and post‐inflammatory hyperpigmentation (PIH). Because eligibility was based primarily on imaging‐defined pigmentary patterns rather than strict diagnostic categories, the study population was considered an imaging‐pattern‐based exploratory cohort.

The conventional‐treatment group (*n* = 38) included patients treated between January 2022 and September 2025 with heterogeneous multimodal laser combinations selected according to lesion characteristics. Treatments involved various combinations of a picosecond Nd:YAG laser (PicoHi; Hironic, Republic of Korea), a long‐pulsed 755‐nm alexandrite laser (Clarity II; Lutronic, Republic of Korea), and a long‐pulsed 532/1064‐nm laser (DermaV; Lutronic, Republic of Korea). This group represented routine multimodal practice rather than a standardized treatment protocol. All procedures were performed by J.Y.B.

The dual‐axis protocol group (*n* = 15) included patients treated between April and December 2025 with the imaging‐guided dual‐axis protocol using the PicoHi picosecond Nd:YAG laser. All procedures were performed by S.J.K.

#### 2.1.1 Exclusion Criteria

Patients were excluded if they had recent intense ultraviolet exposure, active dermatitis or cutaneous infection, current use of photosensitizing medications, pregnancy or lactation, or a history of keloidal scarring.

### 2.2 Dual‐Axis Treatment Protocol (Figure 1)

**FIGURE 1:**
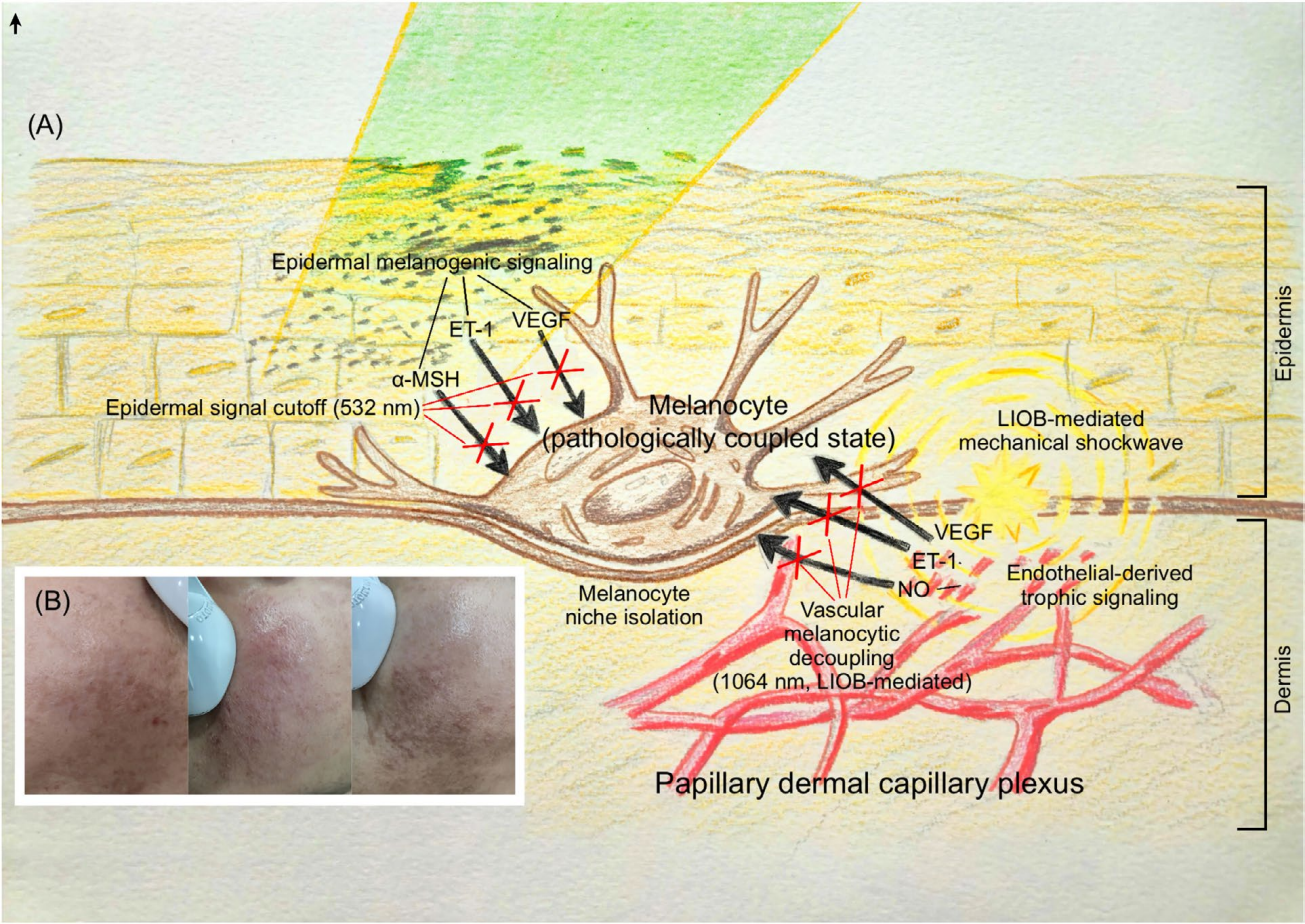
Hypothesis‐level schematic of the imaging‐guided dual‐axis treatment concept. Conceptual schematic and representative clinical images illustrating the proposed dual‐axis treatment framework. The signaling pathways and mechanistic relationships shown are biologically plausible interpretations and were not directly measured in this study. (A) Proposed dual‐axis treatment concept. The first component represents selective 532‐nm treatment of sharply demarcated epidermal‐predominant pigmentation identified clinically and on normal‐light and ultraviolet imaging. Controlled superficial micro‐crusting served as the procedural endpoint, with the aim of reducing the visible superficial pigment burden while allowing epidermal recovery. Epidermal signal cutoff refers to the possibility that selective treatment of this component may also reduce local epidermal influences that support melanogenic activity. The illustrated mediators, including α‐melanocyte‐stimulating hormone, endothelin‐1, and vascular endothelial growth factor (VEGF), are examples reported in previous studies. The second component represents repeated 1064‐nm picosecond irradiation of dermal‐predominant or mixed lesions, with treatment intensity calibrated to individual tissue response within a clinically acceptable sub‐purpuric range. Possible LIOB and associated shockwave effects, attenuation of endothelial‐associated melanogenic support, vascular–melanocytic decoupling, and a signal‐limited melanocyte environment termed melanocyte niche isolation are presented as hypothesis‐level explanations for the coordinated changes in pigment‐ and PL‐associated imaging. (B) Representative tissue responses during 1064‐nm irradiation. Magnified clinical images show transient focal erythema and limited micro‐petechiae immediately after treatment. These responses were used as platform‐specific calibration markers rather than as treatment targets or predefined biological thresholds.

The dual‐axis protocol was designed to address epidermal‐predominant, dermal‐predominant, or mixed pigmentary components separately. Multimodal imaging was used to select treatment targets and calibrate treatment intensity.

Treatments were performed using a picosecond Nd:YAG laser system (PicoHi; Hironic Co. Ltd., Republic of Korea), with nominal pulse durations of 275 ps at 532‐nm and 300 ps at 1064‐nm. Selective 532‐nm irradiation was applied to sharply demarcated superficial epidermal pigmentation, whereas repeated 1064‐nm irradiation was directed at dermal‐predominant or mixed lesions.

For the 532‐nm component, typical treatment parameters were a 4‐mm spot size, a fluence of 0.25–0.30 J/cm^2^, and one to two passes. Controlled superficial micro‐crusting served as the procedural endpoint, with the aim of reducing the visible superficial pigment burden while allowing epidermal recovery.

The lesion‐directed 1064‐nm component was delivered using a 4‐mm spot size, a fluence of 0.7–1.0 J/cm^2^, and a repetition rate of 2 Hz. Each lesion was treated in a single continuous scanning sequence with approximately 50% overlap, corresponding to an effective coverage of approximately 1.5 passes. The repeated 1064‐nm treatment sessions were generally performed at 1–2‐week intervals. Because the number and surface area of the selected lesions varied among patients, the lesion‐directed pulse count was not fixed. Treatment intensity was individualized according to lesion characteristics, skin reactivity, and immediate tissue response within a clinically acceptable sub‐purpuric range. Treatment was calibrated primarily according to transient focal erythema, while limited micro‐petechiae could occur as an acceptable device‐specific tissue response within the intended sub‐purpuric range. Micro‐petechiae were not required as a treatment endpoint or intentionally maximized. The appropriate tissue‐response range may vary across laser platforms according to pulse duration, beam profile, and laser–tissue interaction.

Topical anesthesia was not used, and contact or air cooling was not applied during laser irradiation.

Treatment intensity was reduced, the treated area was restricted, or a subsequent session was postponed when tissue responses exceeded the intended clinical range. No fixed total number of treatment sessions or predefined numerical stopping rule was used.

A standardized post‐procedure supportive‐care protocol was used in the dual‐axis group. Immediately after irradiation, the treated areas received short‐contact application of diluted clobetasol propionate solution, followed by application of a post‐procedure cream, device‐assisted cooling, red‐light LED phototherapy, and a soothing facial mask. Detailed preparation and application procedures are provided in the Supplementary Methods. Post‐procedure care in the conventional‐treatment group was heterogeneous and could not be standardized or reliably reconstructed from the retrospective records.

Further details regarding pulse delivery, lesion coverage, treatment‐response criteria, and treatment‐course decisions are provided in the Supplementary Methods.

### 2.3 Imaging and Outcome Measures

Standardized facial images were obtained using a multimodal imaging system (Mark‐Vu; PSI Plus, Republic of Korea) at baseline, Session 5, Session 9, and the final documented assessment when available. Quantitative analysis was performed using manually defined lesion‐centered regions of interest (ROIs), as detailed in the Supplementary Methods.

The imaging outcomes were:

1. Melanin Index (MI), reflecting pigment‐associated optical features;
2. Ultraviolet (UV) index, reflecting pigmentary features visible on ultraviolet imaging, including subclinical patterning; and
3. Polarized‐light (PL) index, reflecting vascular‐ or inflammation‐associated optical features on polarized‐light imaging.

These indices were treated as imaging‐derived parameters rather than externally calibrated absolute measurements. In particular, the PL index was not considered a direct measure of endothelial function, vascular trophic activity, inflammation, or specific molecular signaling.

### 2.4 Statistical Analysis

The primary analyses evaluated changes in MI, UV index, and PL index from baseline to Sessions 5 and 9. Final assessment values were analyzed separately as exploratory outcomes because the timing of the final evaluation varied among patients.

Within‐group changes were assessed using the Wilcoxon signed‐rank test. Unadjusted between‐group comparisons of absolute baseline‐to‐follow‐up changes were performed using the Mann–Whitney U test. Unadjusted effect estimates were expressed as between‐group differences in median absolute change, with 95% bootstrap confidence intervals based on 5 000 resamples. Exploratory baseline‐adjusted comparisons were conducted using analysis of covariance (ANCOVA), with adjustment for the corresponding baseline index, age, sex, Fitzpatrick skin type, and harmonized clinical diagnosis. Adjusted between‐group differences were reported with 95% confidence intervals and *p*‐values.

Missing data were not imputed; analyses were based on the available cases at each time point. A complete‐case analysis was performed among patients with imaging available at baseline, Session 5, and Session 9. A sensitivity analysis was also conducted after excluding patients with baseline UV indices above the 90th percentile of the overall baseline distribution.

The exploratory composite imaging responder definition required reductions from baseline of at least 20% in MI, 20% in UV index, and 10% in PL index. Separate responder analyses were also conducted for MI, UV index, and PL index using the corresponding individual thresholds. These were considered exploratory imaging‐based outcomes rather than validated clinical response measures.

Intra‐observer directional reproducibility was evaluated in 15 selected patients through two additional manual reassessments of ROI placement and threshold determination.

All tests were two‐sided, and *p* < 0.05 was considered statistically significant. Further details are provided in the Supplementary Methods.

## 3 Results

### 3.1 Baseline Demographic and Clinical Characteristics

A total of 53 patients were included: 15 in the dual‐axis protocol group and 38 in the conventional‐treatment group. Age and sex distributions did not differ significantly between the groups. Baseline MI did not differ significantly between the groups, whereas the conventional‐treatment group had a higher baseline UV index and the dual‐axis protocol group had a higher baseline PL index (Table S1).

Clinical diagnoses included mixed melasma, acquired bilateral nevus of Ota‐like macules (ABNOM), solar lentigines/ephelides, and post‐inflammatory hyperpigmentation (PIH). Clinical diagnosis was recorded descriptively and included as an adjustment variable.

### 3.2 Longitudinal Changes in Quantitative Imaging Indices

At Sessions 5 and 9, the dual‐axis protocol group showed greater reductions from baseline in MI, UV index, and PL index than the conventional‐treatment group (Table 1).

**TABLE 1:** Longitudinal changes in quantitative imaging indices at fixed treatment landmarks and final assessment.

Parameter	Timepoint	Dual‐axis protocol group, *n*	Dual baseline median (IQR)	Dual follow‐up median (IQR)	Dual Δ absolute median (IQR)	Dual Δ% median (IQR)	Conventional‐treatment group, *n*	Conventional baseline median (IQR)	Conventional follow‐up median (IQR)	Conventional Δ absolute median (IQR)	Conventional Δ% median (IQR)	Unadjusted difference in median absolute change (95% CI)	Unadjusted *p*	Adjusted group difference (95% CI)	Adjusted *p*
MI	Session 5	15	83.5 (81.9 to 84.4)	57.5 (53.2 to 63.6)	−25.0 (−31.7 to −16.9)	−30.3 (−37.5 to −20.8)	34	84.1 (82.5 to 85.8)	77.8 (74.9 to 80.2)	−7.1 (−9.3 to −3.6)	−8.5 (−11.2 to −4.3)	−17.92 (−24.60 to −9.50)	< 0.001	−18.67 (−22.37 to −14.96)	< 0.001
MI	Session 9	12	82.6 (81.4 to 83.7)	46.8 (45.7 to 48.9)	−36.3 (−37.2 to −32.0)	−43.7 (−44.9 to −39.6)	38	84.1 (82.9 to 85.8)	76.0 (71.0 to 79.8)	−7.8 (−13.6 to −5.2)	−9.1 (−15.8 to −6.2)	−28.52 (−31.05 to −22.72)	< 0.001	−25.64 (−30.24 to −21.04)	< 0.001
MI	Final^a^	15	83.5 (81.9 to 84.4)	46.8 (45.7 to 48.0)	−36.8 (−38.3 to −33.7)	−44.0 (−45.3 to −41.5)	38	84.1 (82.9 to 85.8)	77.8 (69.8 to 81.1)	−6.6 (−11.7 to −3.5)	−7.7 (−14.2 to −4.1)	−30.17 (−32.55 to −25.88)	< 0.001	−26.16 (−30.89 to −21.43)	< 0.001
UV	Session 5	15	86.2 (85.3 to 86.6)	62.6 (57.4 to 66.7)	−23.4 (−29.9 to −17.1)	−27.2 (−34.8 to −20.3)	34	89.9 (88.0 to 91.3)	84.1 (81.1 to 85.5)	−6.4 (−8.1 to −3.4)	−7.0 (−9.8 to −3.7)	−17.00 (−24.35 to −10.72)	< 0.001	−18.33 (−22.40 to −14.27)	< 0.001
UV	Session 9	12	85.8 (85.0 to 86.4)	50.8 (50.3 to 54.3)	−34.5 (−36.2 to −29.9)	−40.6 (−42.0 to −35.4)	38	90.0 (88.2 to 91.3)	82.2 (78.7 to 85.9)	−7.3 (−12.1 to −5.0)	−8.0 (−14.1 to −5.5)	−27.15 (−30.07 to −20.85)	< 0.001	−25.38 (−29.90 to −20.86)	< 0.001
UV	Final^a^	15	86.2 (85.3 to 86.6)	51.0 (50.2 to 54.1)	−35.0 (−37.3 to −31.4)	−40.9 (−42.6 to −37.1)	38	90.0 (88.2 to 91.3)	83.8 (76.3 to 86.8)	−6.5 (−10.5 to −4.3)	−7.2 (−11.5 to −4.8)	−28.45 (−31.55 to −24.50)	< 0.001	−24.59 (−29.52 to −19.67)	< 0.001
PL	Session 5	15	79.4 (79.4 to 82.9)	69.9 (69.3 to 75.2)	−9.4 (−11.2 to −7.7)	−11.9 (−13.9 to −9.4)	34	78.4 (77.5 to 80.8)	77.2 (76.5 to 79.8)	−1.1 (−2.1 to 0.1)	−1.4 (−2.6 to 0.1)	−8.35 (−10.55 to −6.45)	< 0.001	−7.50 (−9.17 to −5.83)	< 0.001
PL	Session 9	12	79.4 (79.4 to 80.5)	66.3 (65.1 to 66.9)	−14.2 (−15.4 to −12.5)	−18.0 (−19.5 to −15.8)	38	78.5 (77.6 to 79.9)	77.7 (76.4 to 78.5)	−2.0 (−2.8 to 0.1)	−2.6 (−3.4 to 0.1)	−12.20 (−13.88 to −9.73)	< 0.001	−11.81 (−13.49 to −10.12)	< 0.001
PL	Final^a^	15	79.4 (79.4 to 82.9)	66.3 (65.1 to 69.3)	−14.2 (−15.4 to −12.5)	−18.0 (−19.5 to −16.0)	38	78.5 (77.6 to 79.9)	78.3 (77.5 to 78.6)	−0.5 (−2.0 to 1.0)	−0.6 (−2.5 to 1.3)	−13.75 (−15.10 to −11.70)	< 0.001	−12.12 (−13.95 to −10.30)	< 0.001

*Note:* Effect estimates are reported as the dual‐axis protocol group minus the conventional‐treatment group. Negative values indicate greater reduction in the dual‐axis protocol group. Continuous values are shown as median (IQR). Δ absolute = follow‐up value minus baseline value. Δ% = (follow‐up − baseline)/baseline × 100. Unadjusted effect = between‐group difference in median baseline‐to‐follow‐up change; 95% CI calculated by bootstrap resampling. Adjusted group difference was estimated by ANCOVA with the follow‐up index as the dependent variable, treatment group as the main factor, baseline index and age as continuous covariates, and sex, Fitzpatrick skin type, and harmonized clinical diagnosis as categorical covariates. No missing imaging values were imputed; timepoint‐specific available cases were analyzed.

^a^ Final assessment values are exploratory because final assessment sessions varied across patients. Fixed landmark analyses at Session 5 and Session 9 should be treated as primary longitudinal comparisons.

In the dual‐axis protocol group, the median percentage reductions in MI were 30.3% at Session 5 and 43.7% at Session 9, compared with 8.5% and 9.1%, respectively, in the conventional‐treatment group. The corresponding reductions in the UV index were 27.2% and 40.6% in the dual‐axis protocol group vs. 7.0% and 8.0% in the conventional‐treatment group. Median reductions in the PL index were 11.9% and 18.0% in the dual‐axis protocol group, compared with 1.4% and 2.6% in the conventional‐treatment group.

Covariate‐adjusted analyses also showed significant between‐group differences in all three indices. The adjusted differences for MI were −18.67 (95% CI, −22.37 to −14.96) at Session 5 and −25.64 (95% CI, −30.24 to −21.04) at Session 9. The corresponding differences were −18.33 (95% CI, −22.40 to −14.27) and −25.38 (95% CI, −29.90 to −20.86) for the UV index, and −7.50 (95% CI, −9.17 to −5.83) and −11.81 (95% CI, −13.49 to −10.12) for the PL index (all *p* < 0.001). Negative values indicate lower adjusted follow‐up values in the dual‐axis protocol group.

The final assessment showed the same overall direction of change but was considered exploratory because assessment timing varied among patients. Complete‐case analysis restricted to patients with imaging available at baseline, Session 5, and Session 9 yielded findings directionally consistent with the primary analysis (Supplementary Table S2). Sensitivity analysis excluding patients with markedly elevated baseline UV indices also yielded directionally consistent findings across all three indices (Table S3).

### 3.3 Composite and Single‐Index Imaging Responder Analyses

Under the exploratory composite imaging‐responder definition requiring concurrent reductions in the MI, UV, and PL indices, responder rates increased over time in the dual‐axis protocol group, whereas no patient in the conventional‐treatment group met the composite criteria at any evaluated time point. The dual‐axis protocol group also showed higher responder rates according to the MI‐only, UV‐only, and PL‐only criteria (Supplementary Table S4). These responder analyses were considered exploratory imaging‐based outcomes rather than conventional clinical response measures.

### 3.4 Exploratory Imaging‐Response Categories

Exploratory imaging‐response categories were defined using predefined combinations of changes in MI, UV index, and PL index. By Session 9, all available patients in the dual‐axis protocol group were classified as having either a complete or stabilizing composite imaging‐response pattern, whereas all patients in the conventional‐treatment group were classified as having an incomplete composite imaging‐response pattern (Table 2).

**TABLE 2:** Temporal distribution of exploratory imaging‐response categories.

Imaging‐response category	Session 5			Session 9			Final^a^		
	Dual‐axis protocol group (*n* = 15)	Conventional‐treatment group (*n* = 34)	*p*	Dual‐axis protocol group (*n* = 12)	Conventional‐treatment group (*n* = 38)	*p*	Dual‐axis protocol group (*n* = 15)	Conventional‐treatment group (*n* = 38)	*p*
Complete composite imaging‐response pattern	2 (13.3%)	0 (0.0%)		9 (75.0%)	0 (0.0%)		12 (80.0%)	0 (0.0%)	
Stabilizing composite imaging‐response pattern	8 (53.3%)	0 (0.0%)		3 (25.0%)	0 (0.0%)		2 (13.3%)	0 (0.0%)	
Incomplete composite imaging‐response pattern	5 (33.3%)	34 (100.0%)		0 (0.0%)	38 (100.0%)		1 (6.7%)	38 (100.0%)	
Total	15 (100.0%)	34 (100.0%)		12 (100.0%)	38 (100.0%)		15 (100.0%)	38 (100.0%)	
Overall between‐group *p*			< 0.001			< 0.001			< 0.001

*Note:* Categories were assigned using time‐point‐specific available cases; missing imaging values were not imputed. Data are presented as *n* (%). Complete composite imaging‐response pattern: ≥ 20% reduction from baseline in both MI and UV indices and ≥ 15% reduction in PL index. Stabilizing composite imaging‐response pattern: ≥ 20% reduction from baseline in both MI and UV indices and ≥ 10% to < 15% reduction in PL index. Incomplete composite imaging‐response pattern: < 20% reduction in either MI or UV index, or < 10% reduction in PL index. The incomplete composite imaging‐response pattern indicated only that the predefined combined imaging thresholds were not fully satisfied and did not imply an absence of clinically visible improvement. Categories were used only to describe longitudinal imaging trajectories and were not interpreted as directly measured biological states. Analyses used timepoint‐specific available cases; missing imaging data were not imputed. Overall between‐group distributions were compared at each timepoint using the two‐sided Fisher–Freeman–Halton exact test.

Abbreviations: MI = Melanin Index, PL = polarized‐light index, UV = ultraviolet index.

^a^ Final values represent the last documented assessment for each patient and were treated as exploratory because assessment timing varied among patients.

### 3.5 Manual ROI and Threshold Reproducibility Validation

Intra‐observer reproducibility was evaluated in 15 selected cases. The original ROI‐based trajectories were compared with two manual reassessments performed by the same investigator using ImageJ. For each reassessment, ROI boundaries and image‐specific thresholds were newly defined according to the same predefined lesion‐matching and thresholding principles.

Because ROI placement and threshold selection could affect absolute values, reproducibility was assessed by directional concordance rather than numerical agreement. Concordance with the original analysis was 94.4% for the first reassessment and 96.7% for the second.

Directional concordance was consistently high for MI and UV trajectories. Occasional discordance occurred only in PL trajectories with minimal change over time. Thus, the direction of change remained highly consistent across repeated manual assessments, although the analysis did not establish absolute numerical agreement or inter‐observer reliability (Table S5).

### 3.6 Representative Challenging Cases

**FIGURE 2:**
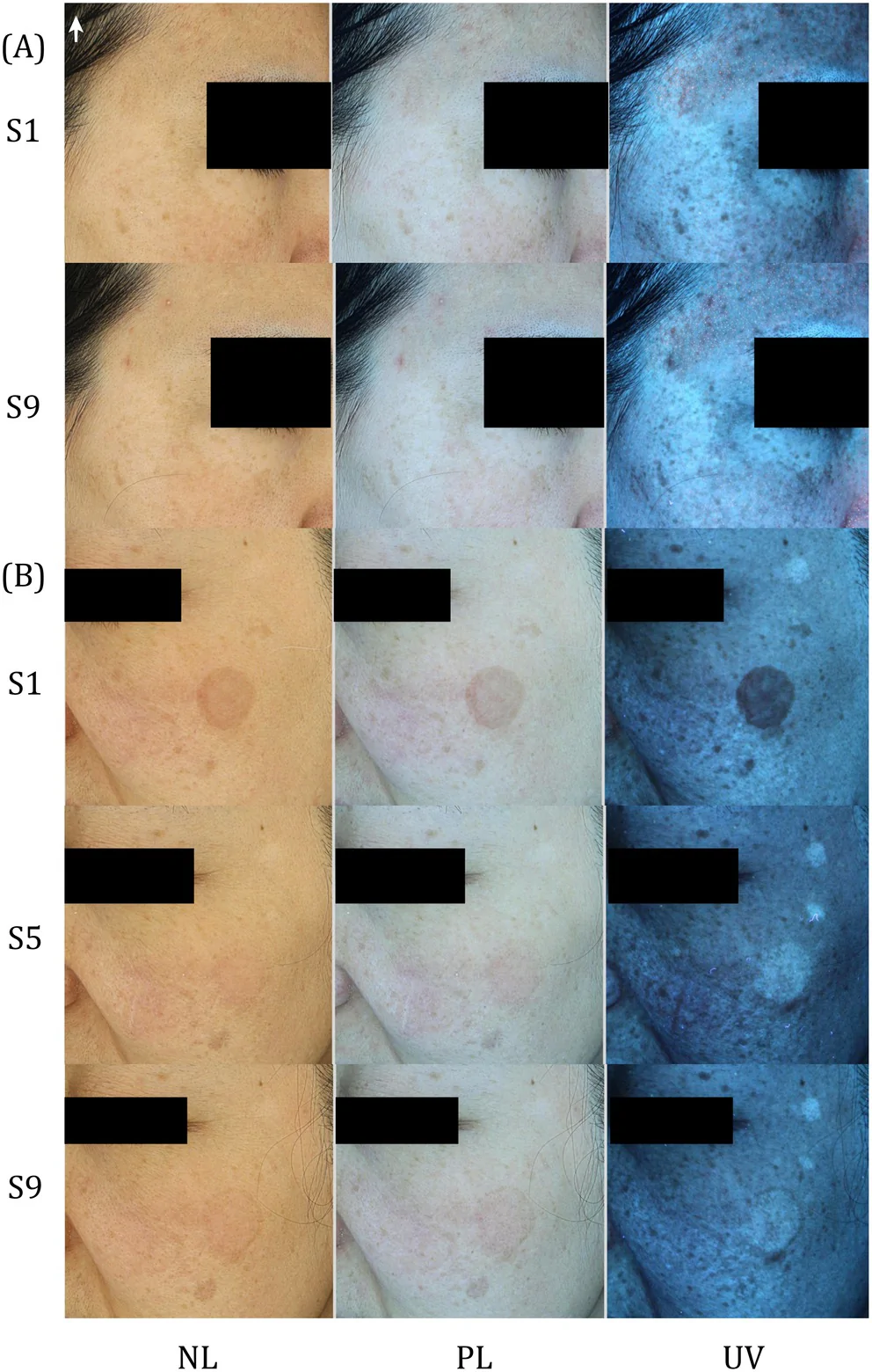
Representative longitudinal multimodal imaging of clinically challenging cases treated with the dual‐axis protocol. Serial multimodal images from two representative cases illustrate how treatment was planned and adjusted according to imaging‐defined pigment distribution and individual tissue response. (A) Clinically ambiguous mixed facial hyperpigmentation with a melasma‐like appearance. In this 42‐year‐old woman, the clinical appearance was suggestive of melasma, whereas ultraviolet imaging showed bilateral symmetrical punctate clusters resembling an ABNOM‐like pigmentary pattern. Treatment was therefore planned according to the imaging‐defined epidermal and dermal pigment components rather than disease nomenclature alone. Pigment‐associated imaging findings gradually improved, with stabilization through Session 9. (B) Reactive phenotype with a strong tendency toward post‐inflammatory hyperpigmentation. This 50‐year‐old woman had marked inflammatory reactivity and delayed pigmentary worsening after previous treatments. Imaging findings and tissue responses were reassessed serially to guide treatment adjustment. Progressive improvement and stabilization were observed through Session 9. For each case, images are shown in normal‐light (NL), polarized‐light (PL), and ultraviolet (UV) modes. NL images demonstrate visible pigment distribution, PL images show polarized‐light optical features, and UV images highlight pigmentary patterning. Corresponding lesion regions were imaged under standardized conditions.

Figure 2 shows two representative cases from the dual‐axis protocol group. In a 42‐year‐old woman with clinically melasma‐like hyperpigmentation, UV imaging revealed bilateral symmetrical punctate clusters suggestive of an ABNOM‐like pattern. Treatment was therefore planned according to the imaging‐defined pigment distribution, and progressive reductions in imaging indices were observed through Session 9.

A 50‐year‐old woman with marked inflammatory reactivity and delayed PIH following previous treatments underwent the dual‐axis protocol. Treatment was adjusted according to serial tissue responses and imaging changes, and progressive improvement and stabilization were observed through Session 9.

### 3.7 Safety

Among the included patients, no serious adverse events, including permanent hypopigmentation, scarring, ulceration, necrosis, or secondary infection, were documented during the available follow‐up period in either group.

In the dual‐axis protocol group, focal erythema and occasional transient focal edema presenting as wheal formation were regarded as expected transient tissue responses. Limited micro‐petechiae could also occur within the intended sub‐purpuric treatment range. Erythema and focal edema generally resolved within 24 h, and no micro‐petechiae persisting beyond 3 days were documented. Superficial micro‐crusting after selective 532‐nm irradiation resolved within approximately 1 week. Transient post‐crusting hypopigmentation or relative lightening was occasionally observed after selective 532‐nm irradiation and resolved during follow‐up, although its incidence and duration were not systematically recorded. No persistent or permanent hypopigmentation was documented.

Patients were informed before treatment that transient erythema or micro‐petechiae could remain visible for approximately 2–3 days. Formal patient‐rated cosmetic acceptability was not assessed; however, no patient discontinued treatment because of these tissue responses. Treatment‐related pain was generally mild, and procedures were performed without topical anesthesia. No acneiform eruption or herpes reactivation was documented in the dual‐axis protocol group.

One highly reactive patient developed delayed PIH‐like worsening after the initial 10 treatment sessions. Subsequent treatment was adjusted according to the evolving tissue response and imaging pattern. Gradual improvement and stabilization were observed after 10 additional sessions. No patient in the dual‐axis protocol group discontinued treatment because of an adverse event.

Because safety assessment was based on retrospective clinical records, the incidence and severity of transient erythema, edema, pain, post‐crusting hypopigmentation, acneiform eruptions, and other minor reactions could not be systematically quantified, particularly in the conventional‐treatment group. No persistent purpura was identified in the available records. Minor adverse events and treatment discontinuation in the conventional‐treatment group could not be reliably assessed, and clinically defined recurrence rates were unavailable.

## 4 Discussion

### 4.1 Theoretical Rationale for an Imaging‐Guided Dual‐Axis Treatment Concept

Conventional treatment of facial hyperpigmentation has largely focused on identifying pigment depth, selecting wavelength‐appropriate treatment parameters, fragmenting visible pigment, and facilitating endogenous pigment clearance [1, 2, 3, 7, 13]. Residual pigmentation is commonly managed with repeated low‐fluence laser toning, which aims to gradually reduce melanocyte activity and melanosome transfer [3, 5]. Although these approaches are effective in many patients, they may be followed by post‐inflammatory hyperpigmentation, recurrence, or a treatment plateau in which further pigment clearance becomes limited [3, 5, 6, 7].

These clinical patterns suggest that persistent or recurrent pigmentation may not always be explained by residual pigment burden alone. Epidermal signals, inflammatory processes, and vascular‐associated factors have all been implicated in maintaining melanocyte activity and pigment persistence [7, 8, 9, 10, 11, 12]. Vascular‐targeted treatment with PDL has also been investigated as an adjunct to pigment‐directed laser therapy in melasma [5]. Therefore, a local tissue environment that continues to support melanogenesis even after partial pigment removal may contribute to recurrence or a treatment plateau.

In the dual‐axis protocol group, reductions in the MI and UV indices were accompanied by reductions in the PL index, whereas this pattern was less consistent in the conventional‐treatment group. Because polarized‐light imaging can enhance the visualization of subsurface optical features associated with erythema and vascular visibility, coordinated changes across all three indices may provide information not captured by visible pigment alone [14, 15]. Conversely, persistent PL‐associated patterns despite varying degrees of pigmentary improvement suggest that pigment‐associated indices alone may not fully characterize the longitudinal multimodal imaging trajectory.

The temporal trajectories were not strictly parallel. Reduction in the MI was already apparent by Session 5, whereas further reductions in the UV and PL indices became more prominent by Session 9. This pattern suggests that visible pigment clearance and changes in UV‐ and PL‐associated imaging features may occur at different rates. However, the present data do not allow determination of whether this temporal separation represents distinct biological stages.

**FIGURE 3:**
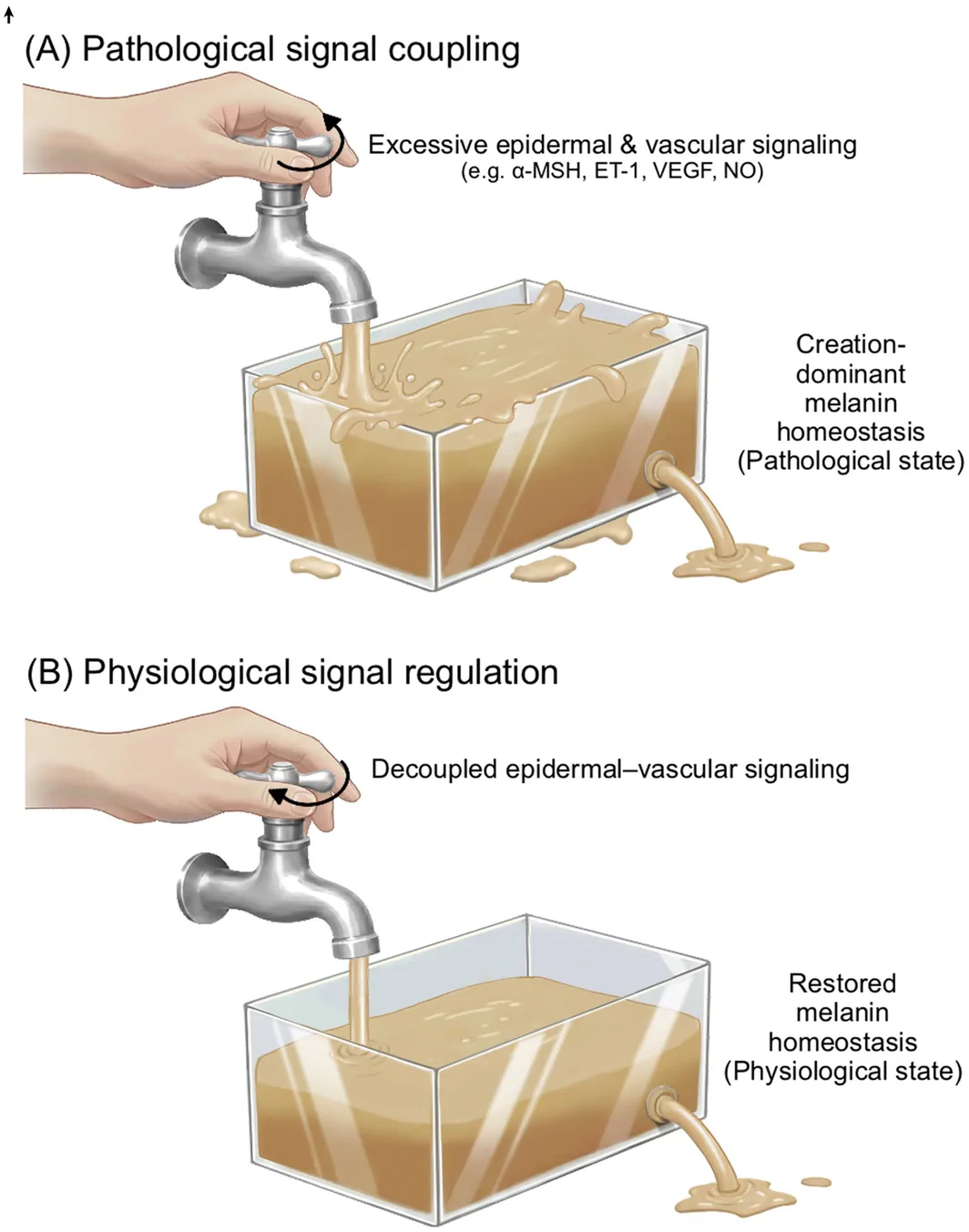
Hypothesis‐level conceptual schematic of melanin homeostasis in facial hyperpigmentation. This fluid‐balance analogy illustrates the proposed relationship between ongoing melanogenic influences and endogenous pigment clearance in persistent facial hyperpigmentation. (A) Hypothesized pathological signal‐supported state. Persistent epidermal, inflammatory, and vascular‐associated influences may support continued melanocyte activation and pigment persistence despite endogenous clearance. The illustrated mediators—α‐melanocyte‐stimulating hormone, endothelin‐1, vascular endothelial growth factor (VEGF), and nitric oxide (NO)—are biologically plausible examples reported in previous studies but were not measured in this study. Creation‐dominant melanin homeostasis is likewise used as a conceptual descriptor rather than a demonstrated biological state. (B) Hypothesized shift toward a more clearance‐favorable state. Attenuation of epidermal and vascular‐associated melanogenic influences is proposed to shift the balance from ongoing pigment production toward endogenous clearance. Epidermal signal cutoff, vascular–melanocytic decoupling, and a shift toward more clearance‐favorable melanin homeostasis represent proposed explanations for the concurrent reductions in MI, UV index, and PL index. These imaging changes provide the empirical context for the model but do not establish the illustrated biological sequence.

In the present study, we used the concept of melanin homeostasis as a hypothesis‐level framework for interpreting these observations. Here, melanin homeostasis refers to the dynamic balance among pigment production, dispersion, and clearance within an interacting epidermal–vascular–melanocytic environment. Mediators such as α‐melanocyte‐stimulating hormone, endothelin‐1, vascular endothelial growth factor, and nitric oxide have been implicated in these interactions and provide biological context for the conceptual framework proposed in this study (8–12; Figure 3). Within this framework, attenuation of persistent melanogenic influences could shift the balance from continued pigment production toward a state more favorable to endogenous clearance.

### 4.2 Dual‐Axis Treatment Strategy Within a Melanin Homeostasis Framework

Within this framework, the dual‐axis protocol was designed to address epidermal‐predominant and dermal‐predominant or mixed pigmentary components separately in patients with recurrent, reactive, or plateau‐type facial hyperpigmentation. Multimodal imaging informed treatment‐target selection and intensity calibration, while serial imaging supported longitudinal documentation and reassessment of the treatment strategy.

The epidermal component involved selective 532‐nm irradiation of sharply demarcated superficial pigmentation evident clinically and on normal‐light and UV imaging. This treatment was intended to reduce the visible epidermal pigment burden while allowing epidermal recovery. Selective removal of the superficial component might also reduce local epidermal influences that support melanogenic activity, a hypothesis‐level effect referred to here as epidermal signal cutoff.

The dermal component involved repeated 1064‐nm picosecond irradiation of dermal‐predominant or mixed lesions. Treatment intensity was adjusted according to lesion characteristics, skin reactivity, and immediate tissue response within a clinically acceptable sub‐purpuric range. Focal erythema and, when present, limited micro‐petechiae served as device‐specific calibration markers. The appropriate response range may vary across laser platforms according to pulse duration, beam profile, and laser–tissue interaction.

The clinical relevance of this approach requires prospective evaluation.

### 4.3 Possible LIOB‐Associated Shockwave Effects

The coordinated imaging changes observed in the dual‐axis protocol group raise the possibility that picosecond Nd:YAG irradiation may exert effects beyond pigment fragmentation alone. One hypothesis‐level mechanism considered to explain these observations is that shockwave‐associated physical interactions might transiently alter the superficial dermal microenvironment.

Under specific irradiation conditions, nanosecond and picosecond laser pulses can produce localized optical breakdown, cavitation, and shock waves [16, 17, 18]. During treatment, transient focal erythema and, in some cases, limited micro‐petechiae were observed, together with visible optical flashes and characteristic acoustic emissions. Although these findings are compatible with photoacoustic tissue interaction, they do not establish that LIOB occurred under the conditions used in this protocol. Rather, these observations provided the basis for considering a possible LIOB‐associated mechanism as a hypothesis‐level interpretation.

If optical breakdown or related shockwave effects did occur, repeated localized perturbation might attenuate vascular or endothelial factors that support melanogenesis within the superficial dermal environment. In the present study, we refer to this hypothetical interruption of sustained upstream support as vascular–melanocytic decoupling. Together with the selective reduction of the epidermal pigment component and its associated melanogenic influences, this process could shift melanin homeostasis from a state favoring melanogenesis toward one more favorable to endogenous pigment clearance.

This model does not require overt destruction of melanocytes or vascular structures. Instead, attenuation of upstream trophic support could create a relatively signal‐limited melanocyte environment, referred to here as melanocyte niche isolation. The concurrent reductions in the MI, UV, and PL indices provide an imaging context in which this model may be considered; however, they do not constitute direct mechanistic evidence. Accordingly, direct confirmation of optical breakdown and histologic and molecular characterization of its tissue effects would be required to evaluate the proposed sequence.

### 4.4 Clinical Rationale for a 1–2‐Week Treatment Interval

Repeated 1 064‐nm treatment sessions were generally performed at intervals of 1–2 weeks. This interval was empirically selected to allow transient erythema, micro‐petechiae, and other immediate tissue responses to resolve while maintaining treatment continuity. It also permitted reassessment of lesion appearance, multimodal imaging findings, and individual tissue response before each subsequent session.

Because different treatment intervals were not compared, the 1–2‐week interval represents a practical clinical schedule rather than a biologically validated optimal treatment window. Whether different treatment intervals influence clinical or imaging outcomes remains unknown.

### 4.5 Permanent Hypopigmentation and Recurrent PL‐Associated Patterns

No permanent hypopigmentation was documented during the available follow‐up period. This observation is relevant in the broader context of laser treatment for facial hyperpigmentation, because repeated low‐fluence 1064‐nm Nd:YAG laser toning has been associated with mottled hypopigmentation [19]. However, melanocyte density, viability, and function were not directly assessed.

Recurrence or partial reappearance of PL‐associated features did not consistently coincide with pigmentary deterioration. In some patients, persistent or recurrent PL patterns accompanied plateau‐type or recurrent pigmentation, whereas in others, partial PL reappearance was not followed by clinical worsening. Accordingly, recurrent PL features did not appear to represent a uniform marker of clinical relapse, and their relationship to vascular–melanocytic re‐coupling remains unresolved.

### 4.6 Study Context, Limitations, and Scope

A principal strength of this study was the longitudinal assessment of lesion‐centered imaging trajectories at fixed treatment landmarks using standardized multimodal imaging. Unlike a cross‐sectional comparison, this approach allowed changes in pigment‐ and PL‐associated indices to be followed concurrently over time. Complete‐case analysis, sensitivity analysis addressing baseline UV imbalance, single‐index responder analyses, and repeated intra‐observer reassessment of ROI placement and threshold selection were used to examine the consistency and directional reproducibility of these patterns.

Several limitations should be considered. First, this was a retrospective, nonrandomized study using a retrospectively identified comparator. The groups differed in treatment period, operator, laser platform, treatment strategy, and adjunctive management. The conventional‐treatment group received heterogeneous multimodal treatments, with some patients also receiving device‐integrated cooling, oral tranexamic acid, or topical depigmenting therapy, whereas adjunctive treatment was more restricted in the dual‐axis protocol group. The contributions of the individual treatment components could therefore not be isolated, and causal attribution of the between‐group differences is limited. Although baseline‐adjusted and sensitivity analyses yielded directionally consistent findings, residual confounding, treatment‐selection bias, operator effects, and period effects remain possible.

Second, the dual‐axis protocol group included only 15 patients across heterogeneous clinical diagnoses. The use of imaging‐defined pigmentary patterns as the principal eligibility criterion reflects real‐world clinical practice but limits diagnosis‐specific inference and subgroup analysis. These findings should therefore be regarded as preliminary and hypothesis‐generating.

Third, the outcomes were investigator‐derived imaging surrogates and did not directly assess vascular function, inflammatory activity, or molecular signaling. Validated pigmentation severity scores, objective colorimetry, blinded physician assessment, patient‐reported outcomes, clinically defined recurrence rates, and histologic or molecular evaluation were unavailable. The proposed biological framework should therefore remain interpretive until examined using direct biological measures.

Fourth, the timing of the final assessment varied among patients, and extended follow‐up beyond the initial treatment course was limited. Missing data were not imputed, and fixed‐landmark analyses used the available cases at each time point. Although the complete‐case analysis yielded directionally consistent findings, attrition‐related bias and differential follow‐up cannot be excluded. Because longitudinal imaging was required for study inclusion, patients who discontinued treatment early or lacked follow‐up imaging may have been underrepresented, particularly in the retrospectively identified conventional‐treatment group. The retrospective clinical records also limited standardized assessment of minor adverse events and treatment discontinuation in the conventional‐treatment group.

Fifth, ROI placement, longitudinal lesion matching, and image‐specific threshold selection were performed manually by an unblinded investigator. Differences in facial orientation, exposure, image brightness, lesion boundaries, ROI size, and threshold selection may have influenced the derived values. Intra‐observer reassessment demonstrated high concordance in the direction of change but did not establish absolute numerical agreement, inter‐observer reliability, or external validity. The results should therefore be interpreted primarily as reproducible directional imaging trends.

Finally, although the protocol was implemented using a picosecond Nd:YAG platform, the broader concept of addressing epidermal‐predominant and dermal‐predominant or mixed pigmentary components, together with their surrounding tissue environment, may not be restricted to a single device. Its application to ruby, alexandrite, pulsed dye, or other laser systems remains theoretical. Differences in wavelength, pulse duration, beam profile, cooling, and tissue interaction require prospective evaluation of each platform before this concept can be generalized.

Future studies should include concurrently treated control groups, standardized clinical and patient‐reported outcomes, blinded and inter‐observer imaging validation, direct characterization of LIOB, direct vascular and biological assessment, and standardized long‐term follow‐up.

## 5 Conclusion

In this retrospective, nonrandomized exploratory cohort of patients with persistent or recurrent facial hyperpigmentation, the dual‐axis protocol was associated with coordinated longitudinal reductions in MI, UV index, and PL index. This coordinated pattern across pigment‐ and PL‐associated imaging domains was less consistently observed in the retrospectively identified conventional‐treatment group. Although the study design does not permit causal comparison of treatment efficacy, the findings provide a rationale for prospectively evaluating whether serial multimodal imaging—including normal‐light, UV, and PL imaging—can assist treatment‐target selection, longitudinal reassessment, and response‐calibrated treatment.

Melanin homeostasis, epidermal signal cutoff, and vascular–melanocytic decoupling remain hypothesis‐level explanations for the observed patterns; the present findings do not establish a specific biological mechanism. Within these limitations, the observed imaging trajectories support prospective evaluation of whether an imaging‐guided dual‐axis approach can selectively address epidermal‐predominant and dermal‐predominant or mixed pigmentary components. Prospective controlled studies using standardized clinical and biological outcomes are needed to validate these imaging findings, determine whether this approach improves clinical outcomes, and clarify the significance of coordinated changes across MI, UV, and PL imaging domains.

## Author Contributions

S.J.K. conceptualized the study, designed the imaging‐guided dual‐axis protocol, performed all procedures in the dual‐axis protocol group, conducted the imaging and statistical analyses, and drafted the manuscript. J.Y.B. performed the procedures in the conventional‐treatment group and contributed to data acquisition. S.J.K. and J.Y.B. critically reviewed the manuscript, and both authors approved the final version.

## Funding

The authors have nothing to report.

## Ethics Statement

The study was conducted in accordance with the principles of the Declaration of Helsinki. Written informed consent for treatment and the subsequent use of clinical data for research purposes was obtained from all participants. All imaging data were fully de‐identified prior to analysis. Institutional Review Board (IRB) approval was waived in accordance with institutional policy, given the retrospective nature of the study and the use of anonymized data.

## Conflicts of Interest

The authors declare no conflicts of interest.

## Supporting information


**Data S1:** Supplementary Methods.

**Table S1:** Baseline demographic and clinical characteristics of the dual‐axis protocol and conventional‐treatment groups.

**Table S2:** Complete‐case analysis at fixed landmark sessions.

**Table S3:** Sensitivity analysis excluding patients with markedly elevated baseline UV indices.

**Table S4:** Composite and single‐index imaging responder rates by treatment group and time point.

**Table S5:** Manual ROI and threshold directional reproducibility validation of longitudinal imaging trajectories.

## Data Availability

The data that support the findings of this study are available from the corresponding author upon reasonable request.
