# Supplementary material for: Longitudinal Imaging Patterns Following an Imaging‐Guided Dual‐Axis Picosecond Nd:YAG Protocol for Facial Hyperpigmentation
Source: J Cosmet Dermatol. 2026 Aug 20;25(8):e71155. doi: 10.1111/jocd.71155 (PMC13494055; doi:10.1111/jocd.71155)
Supplement: Supplementary file 1 [file JOCD-25-e71155-s003.docx]

**Supplementary Methods**

**S1. Inclusion and Exclusion Criteria**

Patients were eligible if they had persistent or recurrent facial hyperpigmentation with coexisting epidermal-predominant and dermal-predominant or mixed pigmentary components identified on standardized multimodal imaging. Eligibility was based primarily on imaging-defined pigmentary characteristics rather than strict clinical disease labels. Specifically, both a clearly identifiable superficial pigmentary component and a deeper dermal-predominant or mixed pigmentary component had to be present within the same lesion or treatment region. Baseline imaging and at least one longitudinal follow-up assessment were required for inclusion.

Clinical diagnoses recorded during routine practice included melasma, acquired bilateral nevus of Ota-like macules (ABNOM), solar lentigines, ephelides, and post-inflammatory hyperpigmentation (PIH). These diagnoses were recorded descriptively and were not used as the primary eligibility criteria. For statistical adjustment, diagnoses were harmonized into four categories: mixed melasma, ABNOM, solar lentigines/ephelides, and PIH.

Patients were excluded if they had recent intense ultraviolet exposure, active dermatitis or cutaneous infection, current use of photosensitizing medications, pregnancy or lactation, or a history of keloidal scarring.

**S2. Dual-Axis Picosecond Nd:YAG Protocol (Figure 1 in the Main Manuscript)**

The dual-axis protocol comprised two lesion-selective components: selective 532-nm irradiation of clearly identifiable epidermal-predominant pigmentation and repeated 1064-nm irradiation of dermal-predominant or mixed pigmentation. Wavelength, treatment area, and treatment intensity were selected according to clinical examination, multimodal imaging findings, apparent pigment depth, and individual skin reactivity.

All dual-axis procedures were performed using a picosecond Nd:YAG laser (PicoHi; Hironic, Republic of Korea). The device delivered nominal pulse durations of 300 ps at 1064 nm and 275 ps at 532 nm.

**S2.1. Selective 532-nm Epidermal Component**

The 532-nm wavelength was applied only to areas with a distinct epidermal-predominant pigmentary component identified by clinical examination and multimodal imaging. These targets were typically sharply demarcated superficial lesions prominent on normal-light and ultraviolet images.

Typical treatment parameters were a 4-mm spot size, a fluence of 0.25–0.30 J/cm², and one to two passes. The procedural endpoint was controlled superficial micro-crusting without excessive erythema, confluent purpura, blistering, or overt tissue injury. Superficial micro-crusting generally resolved within approximately 1 week, with subsequent epidermal recovery observed clinically.

Selective 532-nm treatment could be performed before treatment of the dermal-predominant or mixed component or later during the treatment course if a distinct superficial target remained visible. It was generally avoided in poorly demarcated lesions and predominantly dermal areas without an identifiable superficial component.

**S2.2. Repeated 1064-nm Dermal Component**

The 1064-nm wavelength was applied to dermal-predominant or mixed pigmentary areas identified by clinical examination and multimodal imaging. Typical treatment parameters were a 4-mm spot size, a fluence of 0.7–1.0 J/cm², and a repetition rate of 2 Hz.

Each target lesion was treated using a single continuous lesion-directed scanning sequence with approximately 50% overlap, corresponding to an effective coverage of approximately 1.5 passes. Treatment sessions were generally performed at intervals of 1–2 weeks. Lesion-directed irradiation was confined to target lesions and imaging-defined dermal-predominant or mixed areas; adjacent clinically normal skin without corresponding imaging evidence was excluded from focal treatment. Because the number, size, and distribution of the selected lesions varied among patients, the total pulse count per session was not fixed and could not be consistently determined from the retrospective records.

Treatment intensity was calibrated primarily according to transient focal erythema within a clinically acceptable sub-purpuric range, while avoiding persistent purpura, confluent bruising, blistering, ulceration, necrosis, or other overt tissue injury. Limited micro-petechiae could occur as an acceptable device-specific tissue response within this range but were not required as a treatment endpoint or intentionally maximized. The protocol was intended to deliver the minimum exposure required to reach the intended erythema-based response range rather than a predetermined number of pulses.

**S2.3. Operational Tissue Response and Sub-Purpuric Treatment Range**

Micro-petechiae were clinically distinguished from purpura and bruising. They were defined as small, focal pinpoint hemorrhagic changes appearing immediately after irradiation and resolving within approximately 3 days without persistent discoloration or other evidence of tissue injury. Purpura was defined as larger, confluent, or more persistent vascular discoloration extending beyond the expected transient response. Bruising referred to broader ecchymotic discoloration extending beyond focal pinpoint changes.

With the PicoHi platform, transient focal erythema served as the primary operational marker for calibrating treatment intensity within a clinically acceptable sub-purpuric range. Limited transient micro-petechiae could occur within this range but were not required as a treatment endpoint or intentionally maximized. Neither finding was regarded as a biological threshold or mediator of efficacy. Rather, the observed tissue-response range was specific to the device and treatment conditions used in this cohort.

Treatment settings were adjusted according to lesion characteristics, apparent pigment depth, immediate tissue response, individual skin reactivity, and findings at subsequent visits. The procedural aim was to maintain a controlled tissue response without overt tissue injury.

**S2.4. Treatment Interval**

Repeated 1064-nm treatment sessions were generally performed at intervals of 1–2 weeks. This schedule was selected empirically to allow transient erythema and any limited micro-petechiae, when present, to resolve while maintaining treatment continuity and permitting reassessment before the subsequent session. Because different treatment intervals were not compared, the 1–2-week interval should be regarded as a practical clinical schedule rather than a biologically validated optimal treatment window.

**S2.5. Reporting and Interpretation of Laser Parameters**

Treatment parameters, including fluence, spot size, repetition rate, and irradiation technique, were reported to facilitate clinical interpretation and procedural reproducibility. However, identical numerical settings should not be assumed to produce equivalent tissue responses across different laser platforms.

Picosecond and nanosecond Nd:YAG systems may differ in pulse duration, beam profile, peak power, energy-delivery characteristics, and laser–tissue interaction. The immediate tissue-response range should therefore be interpreted as platform-specific. With the PicoHi system used in this cohort, treatment was calibrated primarily according to transient focal erythema, while limited micro-petechiae could occur as an acceptable response within the intended sub-purpuric range but were not required or intentionally maximized. Application of the reported parameters to other picosecond or nanosecond Nd:YAG systems requires device-specific calibration based on tissue response and tolerability, followed by prospective evaluation of clinical and imaging outcomes.

**S2.6. Treatment-Course Decisions and Discontinuation Criteria**

No fixed number of treatment sessions or predefined numerical stopping rule was used. During each session, treatment intensity was adjusted according to the immediate tissue response. If the response exceeded the intended clinical range—including confluent purpura, excessive or persistent erythema, marked oedema, epidermal disruption, blistering, or intolerable pain—treatment intensity was reduced, the treated area was restricted, or a subsequent session was postponed, as appropriate. No session required premature termination because of these findings.

The overall treatment course was individualized according to serial clinical and imaging findings and patient preference. Two patients elected not to continue after completing 10 sessions; their clinical records documented favorable improvement, although residual cosmetic concerns remained. One patient underwent an additional 10-session course after delayed PIH-like worsening following the initial 10-session course and concluded treatment after 20 total sessions. The remaining patients elected to conclude treatment after clinically favorable improvement documented in routine clinical records. No patient discontinued the dual-axis treatment course because of a treatment-related adverse event.

For the retrospectively identified conventional-treatment group, standardized reasons for treatment completion or discontinuation could not be reliably determined from the available clinical records.

**S3. Adjunctive Therapies and Post-Procedure Care**

Adjunctive management and post-procedure care differed between the two treatment groups.

In the dual-axis protocol group, adjunctive measures were restricted primarily to standardized supportive post-procedure care. Topical anesthesia was not used, and contact or air cooling was not applied during laser irradiation. Oral tranexamic acid and hydroquinone-containing depigmenting creams were not routinely incorporated into the protocol. Patients were advised to maintain appropriate photoprotection.

A standardized supportive-care protocol was applied after each dual-axis treatment session. Cotton pads were saturated with a refrigerated solution prepared by mixing 100 mL of clobetasol propionate solution (Betavate solution; 46.6 mg/100 mL; Korea Pharma Co., Ltd., Republic of Korea), 1,000 mL of distilled water, and approximately five drops of tea tree oil. This preparation corresponded to a nominal final clobetasol propionate concentration of approximately 0.00424%. Immediately after laser irradiation, the saturated cotton pads were applied to the treated areas for 5 minutes. Dr. PPS cream (Careis, Republic of Korea) was then applied, followed by 6 minutes of device-assisted cooling using the Cryocell device (SJ Global, Republic of Korea) and 10 minutes of red-light LED phototherapy using the OMEGA Light device (O’melon, Republic of Korea). Treatment was completed with either a rubber-type facial mask or a sheet mask; the specific mask type and product were not standardized.

In the conventional-treatment group, adjunctive management varied according to the laser platform, lesion characteristics, and the treating physician’s clinical judgment. The DermaV and Clarity II systems provided device-integrated cooling during treatment when applicable. Some patients also received oral tranexamic acid or hydroquinone-containing depigmenting creams as part of routine multimodal management. Post-procedure supportive care was heterogeneous and could not be standardized or reliably reconstructed from the available retrospective records.

Accordingly, the conventional-treatment group represented a heterogeneous multimodal treatment strategy, whereas the dual-axis protocol was implemented with more restricted adjunctive management and standardized post-procedure supportive care. Because adjunctive treatments were not standardized in the conventional-treatment group, their independent contributions to the imaging outcomes could not be isolated.

**S4. Imaging Methodology and ROI Definition**

Standardized facial images were acquired by clinic staff using a multimodal imaging system (Mark-Vu; PSI Plus, Republic of Korea) in normal-light, polarized-light, and ultraviolet modes. Images were obtained at baseline, Session 5, Session 9, and the final documented assessment when available. Image acquisition was performed at a consistent distance and under standardized lighting using the manufacturer’s preset device settings, which were not intentionally changed during the study period. No separate research-specific or external colorimetric calibration was performed. Nevertheless, minor variations in facial orientation, image exposure, and seasonal skin condition were unavoidable during serial acquisition.

Because whole-face averages may not capture subtle or lesion-specific changes, quantitative analysis was performed using lesion-centered regions of interest (ROIs). At baseline, the ROI was placed over the most clinically apparent pigmented area selected as the treatment target for each patient. When bilateral target areas were assessed, the corresponding left- and right-sided ROI values were averaged to generate a single patient-level value for each imaging mode at each time point. At subsequent assessments, the corresponding lesion region was identified by comparison with the baseline image using lesion morphology, anatomical landmarks, and surrounding facial structures. Because facial orientation varied slightly between sessions, the ROI boundary was manually redrawn at each assessment rather than automatically transferred or geometrically overlaid from the baseline image. The same baseline-selected lesion region was followed throughout the longitudinal analysis.

ROI placement, longitudinal lesion matching, and threshold determination were performed manually by the same investigator according to predefined principles. Because of the retrospective design, these procedures were not blinded.

For MI and UV analysis, the normal-light and ultraviolet images within each ROI were converted to grayscale. A single numerical threshold was not applied uniformly to all images because brightness and contrast varied among serial acquisitions. Instead, an image-specific threshold was selected manually according to the visible pigment distribution, lesion boundaries, contrast with surrounding skin, and overall clinical appearance. The number of pixels meeting the pigment-classification threshold was calculated as a proportion of the total ROI area and expressed as a percentage.

The MI and UV indices therefore represented the relative area of clinically guided, threshold-defined pigmentary features within the ROI rather than absolute melanin content or mean pixel intensity. The UV index was used as an imaging surrogate of pigmentary features visible on ultraviolet imaging, including subclinical patterning.

For PL analysis, the polarized-light image within each ROI was separated into red, green, and blue channels. Each channel was converted to 32-bit format, and a red-excess image was generated using the following expression:

R − (G + B)

An image-specific threshold was then selected manually according to the distribution of red-associated optical features, lesion boundaries, and contrast with surrounding skin. Pixels exceeding the selected red-excess threshold were calculated as a proportion of the total ROI area and expressed as a percentage.

The PL index represented the relative area of threshold-defined red-excess optical features within the selected ROI. It was used only as a longitudinal imaging surrogate of vascular- or inflammation-associated optical patterns and not as a direct measure of vascular or inflammatory function.

The analyses focused on within-lesion changes, percentage changes from baseline, and the direction and consistency of trajectories over time. Raw pixel intensities and the selected numerical threshold values were used only for image processing and were not analyzed as outcome variables. The derived indices were intended for longitudinal comparison within the standardized imaging framework and were not regarded as externally calibrated absolute measurements.

Image-specific thresholding accommodated unavoidable differences in brightness and contrast but introduced potential observer-dependent variability. Manual ROI placement and lesion matching were additional possible sources of measurement error. An intra-observer validation was therefore performed to determine whether the direction of change in MI, UV index, and PL index remained consistent when ROIs and thresholds were newly defined in separate measurement rounds.

The derived indices were not validated against pigmentation severity scores, objective colorimetry, histologic findings, or blinded physician global assessment in this retrospective dataset.

**S4.1. Manual Intra-Observer ROI and Threshold Reproducibility Validation**

Intra-observer reproducibility was evaluated in a subset of 15 cases: five from the dual-axis protocol group and ten from the conventional-treatment group. The complete cohort was first analyzed using the original lesion-centered workflow. The same investigator subsequently repeated ROI placement, threshold selection, and image analysis in the selected cases using ImageJ. The first manual reassessment was conducted over approximately 1 month, and the second reassessment began after completion of the first. Each selected case therefore underwent three measurement rounds: the original analysis and two subsequent reassessments.

During each reassessment, ROI boundaries and image-specific thresholds were newly determined according to the same lesion-matching and thresholding principles. Neither the ROIs nor the threshold values were copied or automatically transferred from a previous round. MI, UV, and PL trajectories were then recalculated at the predefined follow-up assessments.

The original analysis focused on the most clinically representative treatment-target area within each lesion. The two validation rounds deliberately used broader lesion-centered ROIs to assess whether the direction of change remained stable despite variation in ROI size and boundary placement. The validation was therefore designed to evaluate directional reproducibility rather than absolute agreement in pixel-derived values.

Directional concordance was defined as agreement in the direction of baseline-to-follow-up change between the original analysis and each manual reassessment. The results are presented in Supplementary Table S5.

Inter-observer reliability was not evaluated because all image analyses were performed by a single investigator.

**S5. Statistical Analysis**

All analyses were performed using SPSS Statistics version 29.0 (IBM Corp., Armonk, NY, USA). Calculations of longitudinal changes and responder classifications were cross-checked using spreadsheet-based recalculation where applicable. Continuous variables were summarized as mean ± standard deviation for approximately normally distributed data and as median with interquartile range for non-normally distributed data. Categorical variables were summarized as number and percentage.

Baseline characteristics were compared using the independent-samples t test for age, the Mann–Whitney U test for imaging indices, and Fisher’s exact test or the Fisher–Freeman–Halton exact test for categorical variables, as appropriate.

**S5.1. Primary Longitudinal Imaging Analysis**

The primary imaging outcomes were MI, UV index, and PL index. Changes from baseline to Session 5 and Session 9 were prioritized as the fixed-landmark longitudinal analyses.

For each index, absolute change was calculated as the follow-up value minus the baseline value. Percentage change was calculated as follows:

$$[(\text{follow-up value}-\text{baseline value})/\text{baseline value}]\times100$$

Thus, negative absolute and percentage changes indicated reductions from baseline.

Within-group changes from baseline were assessed using the Wilcoxon signed-rank test. Unadjusted between-group comparisons of absolute changes were performed using the Mann–Whitney U test. The unadjusted effect estimate was calculated as the median absolute change in the dual-axis protocol group minus that in the conventional-treatment group. Negative median differences therefore indicated greater reductions in the dual-axis protocol group. Corresponding 95% confidence intervals were calculated using percentile bootstrap intervals based on 5,000 resamples performed separately within each treatment group.

Exploratory baseline-adjusted comparisons were performed using analysis of covariance (ANCOVA). For each index and time point, the follow-up value was entered as the dependent variable, treatment group as the main independent variable, and the corresponding baseline value as a covariate. Age was included as a continuous covariate, whereas sex, Fitzpatrick skin type, and harmonized clinical diagnosis were included as categorical covariates. Adjusted effects were reported as estimated between-group differences, calculated as the dual-axis protocol group minus the conventional-treatment group, with 95% confidence intervals and P values. Negative adjusted differences indicated lower follow-up values—and therefore greater baseline-adjusted reductions—in the dual-axis protocol group.

**S5.2. Missing Data and Fixed-Landmark Analysis**

Missing imaging data were not imputed, and analyses were based on the available cases at each time point. Session 5 data were available for all 15 patients in the dual-axis protocol group and 34 of 38 patients in the conventional-treatment group. Session 9 data were available for 12 of 15 patients in the dual-axis protocol group and all 38 patients in the conventional-treatment group.

Final assessment data were available for all patients but were obtained after varying numbers of treatment sessions. These data were therefore retained as exploratory descriptive outcomes and were not included among the primary fixed-landmark analyses.

**S5.3. Complete-Case Analysis**

A complete-case analysis was performed among patients with imaging available at baseline, Session 5, and Session 9. The unadjusted and adjusted procedures used in the primary analysis were repeated in this subset. Unadjusted comparisons were based on Mann–Whitney U tests of absolute changes, with 95% confidence intervals for median differences obtained from 5,000 bootstrap resamples.

This analysis examined whether the principal findings remained directionally consistent when restricted to patients with complete imaging at all fixed landmarks. Results are presented in Supplementary Table S2.

**S5.4. Sensitivity Analysis for Baseline UV Imbalance**

Because the conventional-treatment group had a significantly higher baseline UV index, a sensitivity analysis was performed after excluding patients with markedly elevated baseline UV values. A markedly elevated value was defined as a baseline UV index above the 90th percentile of the overall baseline distribution.

Within-group analyses and unadjusted and baseline-adjusted between-group comparisons were then repeated for Sessions 5 and 9 using the same procedures as in the primary analysis. Results are presented in Supplementary Table S3.

**S5.5. Composite and Single-Index Imaging Responder Analyses**

Imaging responder analyses were performed at Session 5, Session 9, and the final assessment. A composite imaging responder was defined as a patient meeting all three of the following criteria at a given time point: at least a 20% reduction from baseline in MI, at least a 20% reduction in UV index, and at least a 10% reduction in PL index. Single-index responses were defined using the corresponding threshold for each index separately.

The composite definition was used to identify concurrent reductions across pigment- and PL-associated imaging domains. These analyses were exploratory and were not intended as validated clinical response measures; failure to meet the responder criteria did not imply an absence of clinically visible improvement.

Composite and single-index responder rates were compared between groups using Fisher’s exact test. Exact 95% confidence intervals for response proportions were calculated using the Clopper–Pearson method. Results are presented in Supplementary Table S4.

**S5.6. Exploratory Imaging-Response Categories**

Exploratory imaging-response categories were defined to describe combinations of change across the three indices:

1. Complete composite imaging-response pattern: at least a 20% reduction in both MI and UV index and at least a 15% reduction in PL index;
2. Stabilizing composite imaging-response pattern: at least a 20% reduction in both MI and UV index and a 10% to less than 15% reduction in PL index; and
3. Incomplete composite imaging-response pattern: less than a 20% reduction in either MI or UV index, or less than a 10% reduction in PL index.

These categories were distinct from the composite responder definition in Section S5.5. They were designed to differentiate trajectories with a larger PL reduction (≥15%) from those with a more modest PL reduction (10% to <15%) while retaining the same MI and UV criteria. The incomplete composite imaging-response pattern indicated only that the predefined combined imaging thresholds were not fully satisfied and did not imply an absence of clinically visible improvement. The categories were used descriptively and were not interpreted as directly measured biological states.

Between-group distributions were compared using the Fisher–Freeman–Halton exact test because of the small sample size and sparse contingency-table cells.

**S5.7. Statistical Significance and Interpretation**

All tests were two-sided, and P < 0.05 was considered statistically significant. No formal adjustment for multiple comparisons was applied because the analyses were exploratory; P values were therefore interpreted together with effect estimates, confidence intervals, baseline imbalances, missing-data patterns, and sensitivity analyses. Given the retrospective, nonrandomized comparison using a retrospectively identified comparator, between-group estimates were not interpreted causally.
